# Morphohistology of the Digestive Tract of the Damsel Fish *Stegastes fuscus* (Osteichthyes: Pomacentridae)

**DOI:** 10.1100/2012/787316

**Published:** 2012-04-01

**Authors:** Bhaskara Canan, Wallace Silva do Nascimento, Naisandra Bezerra da Silva, Sathyabama Chellappa

**Affiliations:** ^1^Programa de Pós-Graduação em Psicobiologia, Centro de Biociências, Universidade Federal do Rio Grande do Norte, Praia de Mãe Luiza, s/n, 59014-100 Natal, RN, Brazil; ^2^Departamento de Morfologia, Centro de Biociências, Universidade Federal do Rio Grande do Norte, Avenida Salgado Filho 3000, Lagoa Nova, 59072-970 Natal, RN, Brazil

## Abstract

This study investigated the morphohistology of the digestive tract and the mean intestinal coefficient of the damsel fish *Stegastes fuscus* captured from the tidal pools of Northeastern Brazil. The wall of the digestive tract of *S. fuscus* is composed of the tunica mucosa, tunica muscularis, and tunica serosa. The esophagus is short with sphincter and thick distensible wall with longitudinally folded mucosa. Mucous glands are predominant, and the muscular layer of the esophagus presented striated fibers all along its extension. The transition region close to the stomach shows plain and striated muscular fibers. Between the stomach and intestine, there are three pyloric caeca. The intestine is long and thin with four folds around the stomach. The anterior intestine presents folds similar to those of pyloric caeca. The estimated mean intestinal coefficient and characteristics of the digestive system of *S. fuscus* present morphological adequacy for both herbivorous and omnivorous feeding habits.

## 1. Introduction

Characterization of the morphohistology of the digestive tract of fish is fundamental to understand their feeding physiology. Furthermore, it helps in defining the feeding habits of different species. Studies have been conducted on the oral-pharyngeal cavity [1] and structure of the digestive tract [2] of different fish species. Research on the annual population profile of marine fish attempts to understand the feeding dynamics based on stomach content analysis [3, 4]. Although there is an increasing interest in the anatomical and histological aspects of the digestive system of fish, such studies are still scarce for neotropical ichthyofauna. Investigations on food habits using stomach contents and morphological analyses of the structures involved in feeding or those which take part in digestion have helped in interpreting the feeding dynamics and habitat occupation of fish species [5].

The Brazilian damsel fish *Stegastes fuscus* (Cuvier, 1830) (Osteichthyes: Pomacentridae) is commonly found in the coastal reefs of Northeastern Brazil near the equatorial line (06°00′40′′S). This fish is nocturnal, generally inhabiting shallow areas (<8 meters of depth) of biogenic or rocky reef pools. The diet of the adult *S. fuscus* is mainly of algae, but animal items have also been observed [6]. This trophic plasticity is explained by the greater need for protein for rapid growth during the initial phase of life [7].

Despite its small size and low commercial value, *S. fuscus *is an important link in the trophic dynamics of tropical reef ecosystems [8]. The current study was carried out to provide information on the morphohistology of the digestive tract of the damsel fish *S. fuscus*.

## 2. Materials and Methods

### 2.1. Sample Collection

The fish samples were collected from reef tidal pools in Búzios beach, Nizia Floresta, Brazil (06°00′40′′ S; 35°06′33′′ W). In this study, 30 pooled samples of males and females of adult damselfish were used, with mean standard length of 6.95 (±0.94) cm and mean body weight of 10.76 (±2.78) g. Each specimen was ventrally incised to expose the digestive tract which was removed from the coelomic cavity and fixed in 10% formaldehyde.

### 2.2. Procedures

The intestinal coefficient (*C*_i_), which is the ratio of intestinal length (*L*_i_) to body length (*L*_s_), given by *C*_i_ = *L*_i_ · *L*_s_^−1^ was calculated [9]. Intestinal lengths (cm) and body lengths (cm) were obtained from 30 adult specimens of *S. fuscus* (pooled samples of males and females) with mean standard length (*L*_s_) of 6.95 (±0.94) cm. The relation between *L*_s_ and *L*_i_ was verified by correlation analysis [10].

For the morphological description, the general body shape, position and size of the mouth, dentition, type, and shape of the gills rakers were examined.

After 24 hours of fixation, the digestive tract was submitted to microtomy to obtain fragments of the digestive organs: esophagus, stomach (cardiac, fundic, and pyloric regions), pyloric caeca, and the intestine (anterior, intermediate, and posterior portions). Tissue samples were fixed in Bouin's fluid or buffered formaldehyde. The fragments were submitted to routine histological techniques (dehydration, diaphanization, and paraffin embedding) and microsectioned at 5 *μ*m. Samples were stained with haematoxylin-eosin (HE) and periodic acid Schiff (PAS) following routine procedures. The esophagus, stomach (cardiac, fundic, and pyloric regions), pyloric caeca, and the intestine (anterior, intermediate, and posterior portions) were analyzed through transverse and longitudinal sections. Additionally, a sagittal section of the whole tract aided in the identification of transition regions. For anatomical analysis, a stereoscope (PZO Labimex) was used. For the slide microphotography procedure, an Olympus B202 microscope coupled to a Nikon DXM 1200 digital camera was used, and the images were processed using Nikon ACT-1 software.

For histological descriptions, attention was given to the thickness, length, and presence of folds, valves, and sphincters in the digestive organs. The mucosal epithelial structure, cell types, occurrence of glands, characterization of the submucosa, and distribution of granular cells were considered. The organization of the tunica muscularis and tunica serosa was also taken into account.

## 3. Results

### 3.1. Gross Anatomy

**Figure 1:**
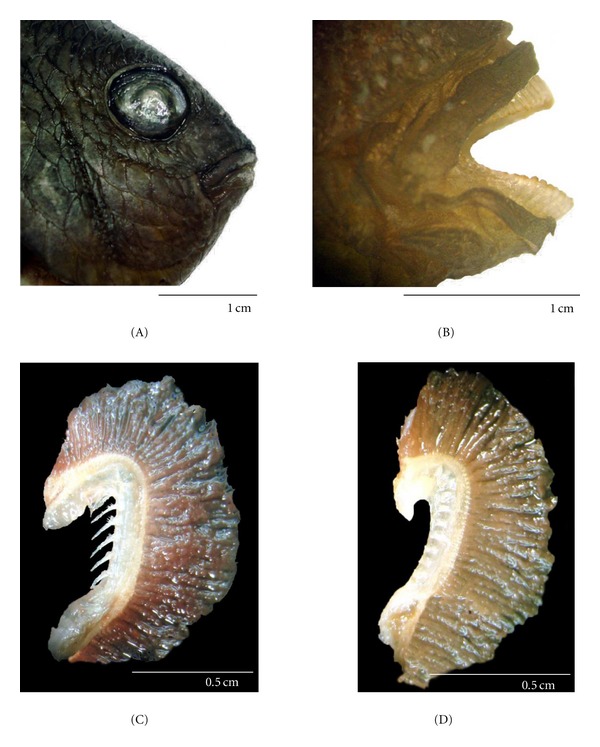
Lateral view of the head of* S. fuscus: *(A) mouth closed; (B) mouth open showing the teeth; (C) the first branchial arch showing the gill rakers; (D) the second branchial arch.

*S. fuscus* has a laterally long body with a slightly protractile terminal mouth (Figure 1(A)). The upper and lower lips are comprised of a stratified epithelium, with nonserrated incisive teeth set in a single row on the jaws (Figure 1(B)). Each gill chamber has four gill arches, the first arch possesses long thin gill rakers (Figure 1(C)), while the other arches possess unadorned gill rakers (Figure 1(D)).

**Figure 2:**
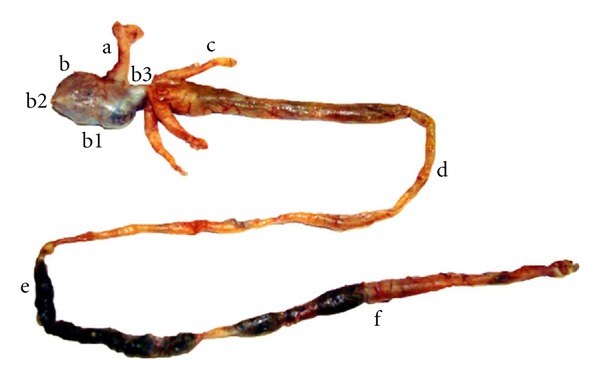
Structures of the digestive tract of *S. fuscus*: (a) esophagus, (b) stomach, (b1) cardiac stomach, (b2) fundic stomach, (b3) pyloric stomach, (c) pyloric caeca, (d) anterior intestine, (e) midintestine, and (f) posterior intestine.

*S. fuscus* presents a wide but short tubular esophagus that runs from the posterior end of the pharynx to the anterior cardiac region of the stomach. The sac-like stomach is divided into the cardiac, fundic, and pyloric regions. The pyloric region extends towards the beginning of the intestinal tube. At the junction of the stomach with the intestine, there are three pyloric caeca. The intestine has anterior, medium, and posterior portions (Figure 2). Ventrally, the rectum ends at the anus situated in front of the anal fin.

### 3.2. Histology of the Digestive Tract

#### 3.2.1. Esophagus and Stomach

**Figure 3:**
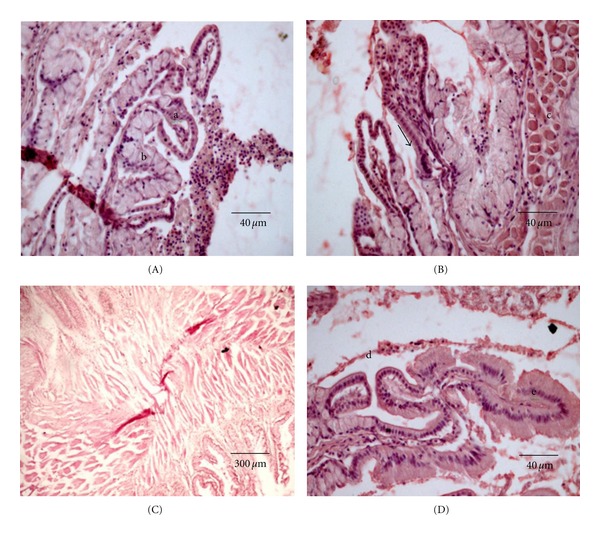
Esophagus of *S. fuscus.* (A) Mucosa with folds consisting of (a) cubic epithelium and (b) mucous glands; (B) mucosa with a tubular mucous secretory gland (arrow) and (c) muscle layer consisting of striated skeletal muscles; (C) esophagus sphincter; (D) esophagus-stomach region showing cubic epithelium of esophagus (d) and cylindrical epithelium of the stomach (e).

The esophagus has concentric tissue layers of tunica mucosa, submucosa, and tunica muscularis (which is made of two layers, an inner circular and an outer longitudinal). The mucosal epithelium shows goblet cells near to the surface, and club cells occur at the basal region of the epithelium. The mucosa has numerous small longitudinal folds lined by stratified epithelium containing numerous mucous cells (Figure 3(A)) followed by lamina propria filled with collagen fibers. Its thick and distensible wall has a large number of striated skeletal muscle fibers, without the presence of smooth muscle fibers (Figure 3(B)). The submucosa layer is formed of dense connective tissue lacking glands. The transition from esophagus to stomach is sharp. The presence of an esophageal sphincter (Figure 3(C)) and a gradual substitution of the simple cubic epithelium of the esophagus by the simple cylindrical epithelium of the stomach (Figure 3(D)) is evident in the transitional region.

**Figure 4:**
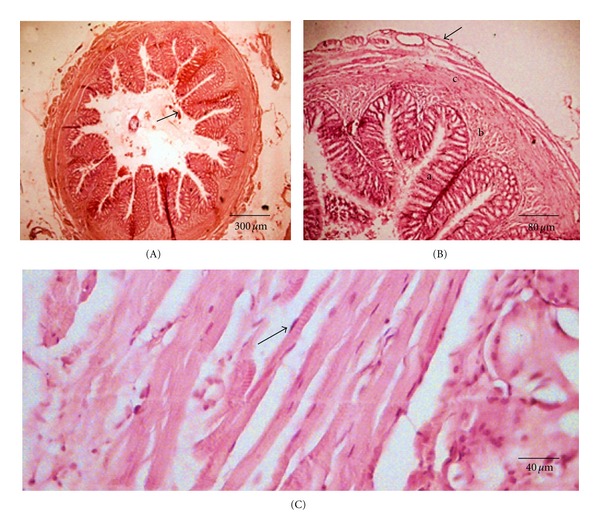
Cardiac stomach of *S. fuscus*. (A) Primary esophagic folds with cardiac mucosa (arrow); (B) gastric glands in stomach lamina propria: (a) conjunctive tissue which forms the submucosa (b), muscular layer (c), and arrow indicating serosa; (C) muscular layer with arrow indicating striated skeletal muscle fibers.

**Figure 5:**
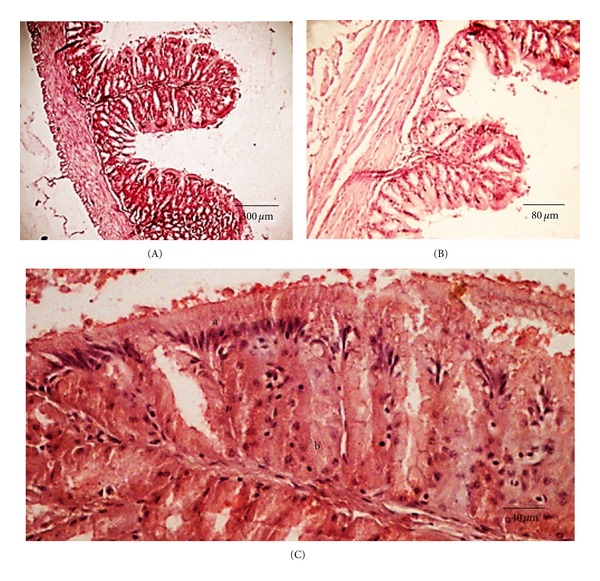
Fundic stomach of *S. fuscus *(A and B) stomach folds lower in comparison to the cardiac region; (C) simple secretory cylindrical epithelium (a) and simple tubular gastric glands (b).

The sac-like stomach of *S. fuscus* is divided into the cardiac, fundic, and pyloric regions. In the cardiac, region the folds are lined with a simple cubic epithelium and lamina propria that branch off and form small secondary folds (Figure 4(A)). Mucous glands predominate in the lamina propria, which produce mucous to facilitate the passage of food. The muscular mucosa and submucosa are not evident. However, the muscular layer with the longitudinal tunica and the circular tunica is well developed. The last layer is a serous membrane with loose connective conjunctive tissue covered by a mesothelium. The gastric glands (Figures 4(B) and 5(C)), when present, are always located in the lamina propria and not in the submucosa.

The division of the stomach into three regions is based on the distribution of gastric glands and the presence and thickness of the mucosal folds (Figures 5(A) and 5(B)). The mucosa of the pyloric stomach consists of a simple mucous secretory prismatic epithelial lining where the glands are formed by mucous secreting cells (Figure 5(C)). Gastric pits are located in the cardiac region and there is a gradual loss of glands in the fundic region. Three pyloric caeca are connected to the intestine after the pyloric region.

**Figure 6:**
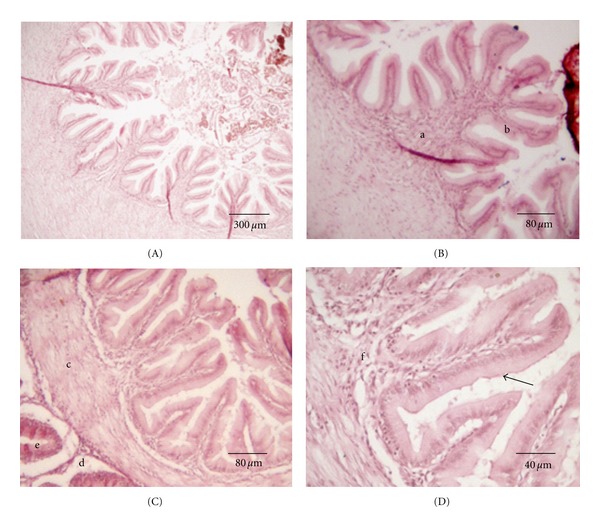
Pyloric stomach of *S. fuscus: *(A) pyloric folds in the stomach mucosa; (B) pyloric mucosa, primary fold (a) with evaginations of the lamina propria, with nonglandular secondary folds (b); (C) pyloric with muscles (c) serosa (d) with conjunctive of pyloric caeca (e); (D) pyloric folds with simple mucous secretory cylindrical epithelium (arrow) and nonglandular lamina propria (f).

The pyloric region contains only the lamina propria, which originates along with the primary and secondary epithelial folds in the stomach (Figure 6(A)). The pyloric region is a nonglandular area (Figures 6(B), 6(C), and 6(D)). The muscularis mucosa is not well developed; however, the presence of stomach submucosa in the cardiac and fundic regions is evident (Figure 6).

The muscle layer of the stomach has an inner layer of smooth muscle fibers set in a circular arrangement and an outer layer of longitudinally placed fibers. The cardiac region of the stomach contains scattered skeletal striated fibers that may have originated in the esophageal region (Figures 4(A) and 4(C)). The most developed muscle layer is found in the pyloric region. The serosa along the whole stomach is a thin dense connective tissue limited by a single layer of flat mesothelial cells. In the pyloric stomach, the serous membrane is continuous with the connective tissue of the serous sac that houses the pyloric caeca (Figure 6(C)).

#### 3.2.2. Pyloric Caeca

**Figure 7:**
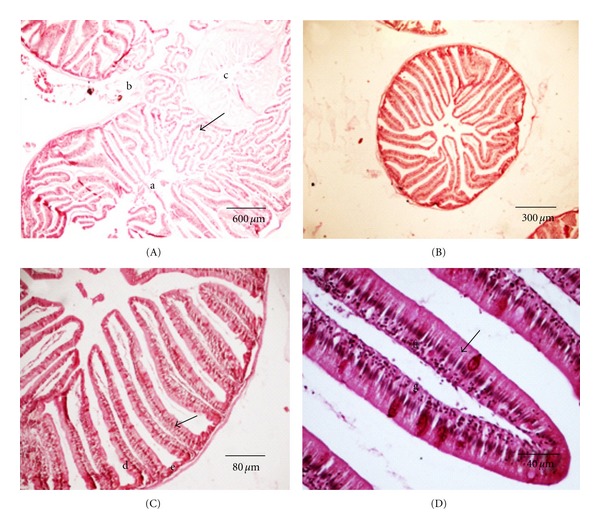
Pyloric caeca of *S. fuscus*. (A) Insertion of caeca (a) in bolsa serosa (b) common to pyloric stomach (c), conjunctive of serosa originating the lamina propria of caeca (arrow); (B) pyloric caeca with mucosa containing numerous folds; (C) caeca with simple cylindrical epithelium showing goblet and absorptive cells (arrow) and lamina propria (d) supported on fine muscle layer (e); (D) cecal fold with epithelium (f) nonglandular lamina propria (g) and goblet cells (arrow).

Three long caeca are present after the pyloric sphincter at the junction of the stomach with the intestine (Figure 7(A)). The mucosa with long folds (Figure 7(B)) is comprised of a single-layered epithelium with cylindrical cells (Figure 7(C)) and lamina propria of loose connective tissue containing small vessels without glands (Figure 7(D)). An epithelium containing prismatic cells (absorptive cells responsible for nutritional absorption) was observed. In the spaces, mucous-producing goblet cells were found, which lubricate the caecal and the intestinal wall (Figures 7(A) and 7(D)). There is no submucosa, instead a thin circular layer of smooth muscle is present.

#### 3.2.3. Intestine

**Figure 8:**
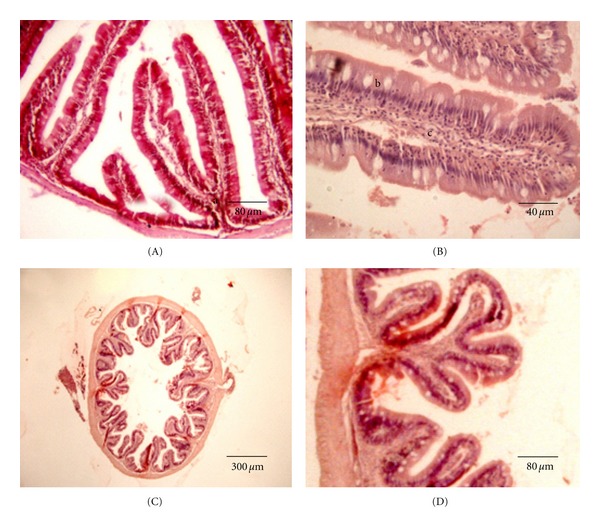
Intestine of *S. fuscus*. (A) Anterior intestinal mucosa, with ramified folds (a); (B) intestinal fold constituted by a simple cylindrical epithelium with goblet and absorptive cells (b) and lamina propria of conjunctive; (C) posterior intestine with mucosa showing short intestinal folds and (D) intestinal folds situated above the thick muscles.

The intestine is moderately long and thin which is typical of herbivorous fish. It forms four folds around the stomach, with their circumvolutions joined by adipose tissue. The estimated mean intestinal coefficient is 2.18 ± 0.15. Though *S. fuscus* does not exhibit a very long intestine, the long folds observed from the caeca to the intestine could be responsible for efficient nutritional absorption. The anterior intestine has folds similar to those identified in the pyloric caeca, but they branch off (Figure 8(A)). The epithelium is cylindrical with a greater number of goblet cells (Figure 8(B)); however, glands are absent in the mucosa and submucosa. After the lamina, propria is the muscle layer, consisting of an inner circular tunica and an outer longitudinal tunica of smooth muscle tissue. The last layer is the serous membrane. In the distal part of the intestine, the folds are smaller, but the muscle layer is thicker (Figures 8(C) and 8(D)).

## 4. Discussion

Fishes feed on the various resources found at different depths of water and their digestive tract descriptions and feedings habits provide useful ecological and biological information. *S. fuscus *is a territorial fish that defends a territory (used for shelter, feeding, or reproductive purposes) and displays intra and interspecific aggressiveness toward other herbivores fish and invertebrates [11]. It has a slightly protractile terminal mouth with flat nonserrated incisive teeth which are used for cropping macroalgae in the reef pools. The diet of the adult *S. fuscus* is mainly (70%) of macroalgae and 30% is of animal items [12].

The gape of the mouth is proportional to the size of the food item consumed. The position of the mouth, type of dentition, degree of mouth protractility, and the type of the gill rakers are related to the feeding habits of fish [13, 14]. It is generally assumed that gill rakers function as a sieve to extract food organisms from the water. A relation exists between gill raker morphology and trophic status of the fish. In the present study, it was observed that in *S. fuscus *only the first pair of gills have long gill rakers which contribute to retain small food items. The other gill rakers were unadorned and seem not to have any role in straining food. There is a correlation between the number of gill rakers, the space between them, their length, and the feeding habits of fish [15].

 Generally, in the anterior part of the esophagus, the skeletal striated fibers predominate, and the posterior part is composed of smooth muscle fibers. However, in *S. fuscus,* the esophageal muscle layer is composed of striated fibers over its full length. There are smooth and striated muscle fibers near the transition region of the stomach. The esophageal muscle layer consists of a longitudinal inner sublayer and a circular outer sublayer [16, 17]. A similar pattern was observed for *S. fuscus*.

In fish, the stomach is an organ with varied shape and structure, depending on their food habits and in some species it may be absent. The stomach in most fish is a dilation of the digestive tube, where food is retained for the required time for acid digestion. The inner mucosa of fish stomach forms winding longitudinal grooves that disappear when the stomach expands with the entry of food. The cardiac and fundic regions are glandular, and the pyloric region is nonglandular. It was also proven in this study that there is no distinction between gastric gland cell types, as occurs in mammals. In a study of the gastric mucosa of *Tilapia* sp., no difference was found between parietal and zymogenic glandular cells, assuming that acid chloride and pepsinogen are secreted by the same cell [18].

The length of the intestine is related to feeding habits, more so in iliophagous, herbivorous, and omnivorous and to a lesser extent in carnivorous and insectivorous fish species. Generally, the intestine is a tubular organ, through which food passes and where alkaline digestion and nutrient absorption occur. The intestinal coefficient is used widely to classify species into trophic categories. According to this pattern, high *C*_i_ values are associated with herbivorous species, whereas intermediate values are associated with omnivorous, and low values with carnivorous fish species. A functional explanation for the long intestine of herbivorous fish species is that some components of the diet are slow to be digested and require both a longer time and more extensive exposure. *S. fuscus *consumes large amounts of plant material together with some small animal items [6].

In teleost fish, there is no separation between the small and large intestine; there is just an undifferentiated tube. The intestinal portions that contain more complex mucosa are generally involved in absorptive processes. The intestinal caeca are blind finger-like structures located in the pyloric region. These structures increase the absorption area of the intestine. The intestinal quotient value calculated for *S. fuscus *is comparable to that reported [14]. Many fish species have flexible diets, using the most readily available resources in the environment, and few are strictly carnivorous or herbivorous. *S. fuscus* is a herbivorous fish with morphological adaptations for omnivorous feeding habits.

## 5. Conclusions

This study confirms that the digestive tract of *S. fuscus* has morphological adequacy for both herbivorous and omnivorous feeding habits, thus indicating its important position in the food chain and consequent role in the ecosystem dynamics. Well-developed incisive teeth enable them to tear plant material and prey on substrate-associated organisms. Food ingestion is aided by an expandable esophagus, and the mucous secretion aids in lubrication of food. The determination of the cellular structures along the digestive tract helps to equate all feeding possibilities for this species which sometimes are insufficiently answered through direct observations. Further investigations, such as electron microscopic studies associated with histochemical analyses, would be useful.
